# Tubastatin A, an HDAC6 inhibitor, alleviates stroke-induced brain infarction and functional deficits: potential roles of α-tubulin acetylation and FGF-21 up-regulation

**DOI:** 10.1038/srep19626

**Published:** 2016-01-21

**Authors:** Zhifei Wang, Yan Leng, Junyu Wang, Hsiao-Mei Liao, Joel Bergman, Peter Leeds, Alan Kozikowski, De-Maw Chuang

**Affiliations:** 1 School of Basic Medicine, Shanghai University of Traditional Chinese Medicine, Shanghai 201203, P.R. China; 2 Molecular Neurobiology Section, National Institute of Mental Health, National Institutes of Health, Bethesda, Maryland 20892-1363, USA; 3 Drug Discovery Program, University of Illinois at Chicago, Chicago, Illinois 60612, USA

## Abstract

Histone deacetylase (HDAC) 6 exists exclusively in cytoplasm and deacetylates cytoplasmic proteins such as α-tubulin. HDAC6 dysfunction is associated with several pathological conditions in the central nervous system. This study investigated the beneficial effects of tubastatin A (TubA), a novel specific HDAC6 inhibitor, in a rat model of transient middle cerebral artery occlusion (MCAO) and an *in vitro* model of excitotoxicity. Post-ischemic TubA treatment robustly improved functional outcomes, reduced brain infarction, and ameliorated neuronal cell death in MCAO rats. These beneficial effects lasted at least three days after MCAO. Notably, when given at 24 hours after MCAO, TubA still exhibited significant protection. Levels of acetylated α-tubulin were decreased in the ischemic hemisphere on Days 1 and 3 after MCAO, and were significantly restored by TubA. MCAO markedly downregulated fibroblast growth factor-21 (FGF-21) and TubA significantly reversed this downregulation. TubA also mitigated impaired FGF-21 signaling in the ischemic hemisphere, including up-regulating β-Klotho, and activating ERK and Akt/GSK-3β signaling pathways. In addition, both TubA and exogenous FGF-21 conferred neuroprotection and restored mitochondrial trafficking in rat cortical neurons against glutamate-induced excitotoxicity. Our findings suggest that the neuroprotective effects of TubA likely involve HDAC6 inhibition and the subsequent up-regulation of acetylated α-tubulin and FGF-21.

Histone deacetylases (HDACs) are promising therapeutic targets for treating neurodegenerative diseases and stroke via epigenetic and non-epigenetic mechanisms^1^,^2^. Pan and isoform-specific HDAC inhibitors exert beneficial effects in diverse brain disorders including ischemic stroke^1^,^3^. HDAC6 is a unique member of the class IIb HDACs, with two catalytic domains and predominantly cytoplasmic localization^4^. This HDAC isoform regulates various cellular processes, including microtubule-based transport, cell motility, endocytosis, cell migration, autophagy, and aggresome formation, by deacetylating non-histone proteins, such as α-tubulin, cortactin, and heat shock protein 90 (HSP90)^5^.

Accumulating evidence suggests that HDAC6 is a promising target for neuroprotection and regeneration in the treatment of central nervous system (CNS) diseases^6^. For instance, its protein levels are significantly increased in the postmortem cortex and hippocampus of Alzheimer’s disease patients^7^. In the postmortem striatum of Huntington’s disease patients, a dramatic decrease was observed in levels of acetylated α-tubulin, a major substrate of HDAC6, suggesting a microtubule-based transport deficit in this disease^8^. Notably, HDAC6 inhibition can reverse this deficit by increasing acetylation of α-tubulin, facilitating the recruitment of molecular motors to microtubules, and promoting the vesicular transport and release of brain-derived neurotrophic factor (BDNF)^8^. Brain ischemia can increase HDAC6 expression levels and disrupt microtubule-based transport by depolymerizing microtubules and decreasing motor proteins levels^9^,^10^,^11^. The disturbance of microtubule-based transport may disrupt mitochondrial transport between the neuronal cell body and the axon/dendrites, and further cause mitochondrial dysfunctions and subsequent cell death^9^. Selective knockdown of HDAC6 by shRNA has been shown to protect mouse cortical neurons from oxygen and glucose deprivation, an *in vitro* cerebral ischemia model^11^.

Tubastatin A (TubA) is a potent and highly selective HDAC6 inhibitor with an IC_50_ of 15 nM and more than 1000-fold selectivity toward all other isoforms except HDAC8 (57-fold)^12^. TubA has a two-hour plasmatic half-life and an AUC_brain_/AUC_plasma_ ratio of 0.18 in mice^13^. In mice, daily intraperitoneal (i.p.) injection of 25 mg/kg TubA for 20 days does not affect gross brain morphology, brain/body mass, liver enzyme measurements, nor kidney function^14^. In several experimental models of central and peripheral nervous diseases, TubA has exhibited neuroprotective effects. Specifically, it rescued neuronal death from oxidative stress induced by homocysteic acid, reversed axonal loss in a mouse model of Charcot-Marie-Tooth disease, improved cognitive deficits in a mouse model of Alzheimer’s disease, and facilitated BDNF trafficking in hippocampal neurons in a mouse model of Rett syndrome^12^,^14^,^15^,^16^.

The present study investigated the possible beneficial effects of TubA in a rat model of ischemic stroke and an *in vitro* model of excitotoxicity. A recent study from our laboratory found that fibroblast growth factor-21 (FGF-21), a metabolic regulator, is expressed in brain neurons and is neuroprotective against glutamate-induced excitotoxicity by targeting the Akt signaling pathway^17^. Therefore, the present study also investigated whether FGF-21 is involved in the neuroprotective effects of TubA.

## Results

### Post-ischemic treatment with TubA improved functional recovery, reduced brain infarct volume and ameliorated neuronal cell death in MCAO rats

**Figure 1:**
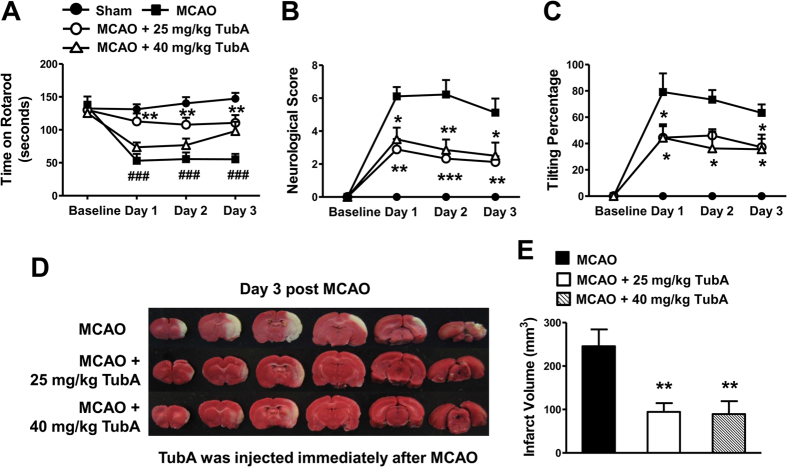
Post-ischemic treatment with TubA (25 and 40 mg/kg) improved functional recovery and reduced brain infarct volume in the middle cerebral artery occlusion (MCAO) model in rats at least three days after ischemia. (**A**) MCAO rats receiving TubA spent much longer time on an accelerating rotarod compared with the untreated MCAO group. The increased neurological deficit score (**B**) and body tilting percentage (**C**) in MCAO rats were markedly attenuated by TubA treatment. (**D**) TubA reduced brain infarct volume in MCAO rats on Day 3 after ischemia. Brain infarction was detected by TTC staining and shown in the white areas. (**E**) Quantification of infarct volume. ^###^*P* < 0.001 compared with sham control; *P < 0.05, **P < 0.01, ***P < 0.001 compared with MCAO group; n = 8 per group.

The time that rats were able to stay on an accelerating rotarod was dramatically reduced one day after MCAO, and this reduction persisted for at least three days (Fig. 1A). When given immediately after ischemia, TubA at 25 mg/kg robustly prolonged the rotarod retention time starting from Day 1, an effect that lasted for at least three days. However, TubA at a higher dosage of 40 mg/kg only had a tendency to ameliorate this motor function deficit. MCAO also induced severe neurological deficits and increased body asymmetry, as indicated by body tilting percentage, starting from Day 1 post MCAO (Fig. 1B,C). At both 25 and 40 mg/kg, TubA markedly ameliorated neurological deficits and reduced body tilting percentage on Day 1 or 3 after ischemia.

Severe brain infarction was observed on Day 3 after ischemia. As shown in Fig. 1D,E, post-ischemic treatment with TubA at both doses significantly reduced mean brain infarct volume from 246 mm^3^ to 94 mm^3^ (25 mg/kg) and 89 mm^3^ (40 mg/kg). In the pilot studies, 25 and 40 mg/kg TubA produced similar effects on HDAC6 inhibition as shown by increasing acetylation of its specific substrate α-tubulin in the cortex of naïve rats (data not shown). Compared with 25 mg/kg, TubA at 40 mg/kg did not produce significantly better effects in neurological deficit score, body asymmetry and brain infarction; furthermore, the lower dose provided a better effect in the rotarod performance of MCAO rats. These results suggested that 40 mg/kg TubA’ s inhibition on HDAC6 may have reached a plateau stage. Therefore, 25 mg/kg was used in the subsequent *in vivo* experiments.

**Figure 2:**
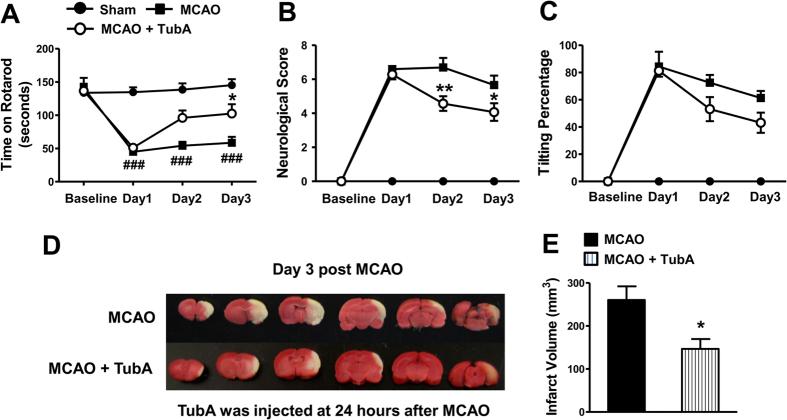
TubA (25 mg/kg) administered at 24 hours after ischemic onset significantly improved functional recovery and reduced brain infarct volume in the middle cerebral artery occlusion (MCAO) model in rats at least three days after ischemia. Functional deficits were evaluated by Rotarod test (**A**), neurological deficit score (**B**), and body tilting test (**C**). (**D**) Brain infarction was assessed by TTC staining on Day 3 after MCAO. (**E**) Quantification of infarct volume. ^###^*P* < 0.001 compared with sham control; *P < 0.05, **P < 0.01 compared with MCAO group; n = 8 per group.

**Figure 3:**
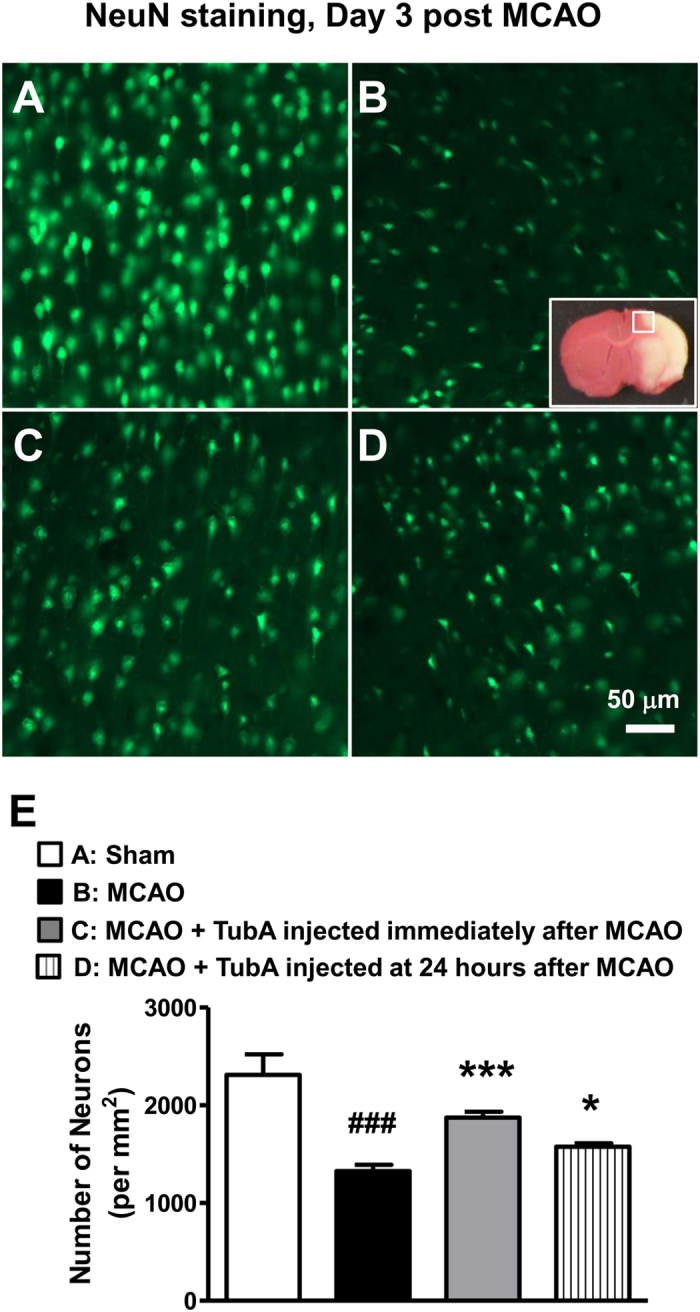
TubA (25 mg/kg) ameliorated neuronal cell death in the ischemic cortex on Day 3 after ischemia. As detected by NeuN staining, middle cerebral artery occlusion (MCAO) induced severe neuronal cell death (**B**) in the ischemic cortex, compared with sham control (**A**). TubA, given immediately (**C**) or at 24 hours (**D**) after MCAO significantly increased neuron survival. (**E**) Quantification of number of neurons per mm^2^ in the ischemic cortex. ^###^*P* < 0.001 compared with sham control; *P < 0.05, ***P < 0.001 compared with MCAO group; n = 6 per group.

Notably, when TubA was given 24 hours after ischemic onset, the treatment still significantly improved rotarod performance on Day 3, and decreased neurological deficit score on Days 2 and 3 (Fig. 2A,B). It also tended to decrease body tilting percentage on Days 2 and 3 (Fig. 2C). Furthermore, it markedly reduced brain infarct volume from 260 mm^3^ to 147 mm^3^ on Day 3 (Fig. 2D,E). As detected by NeuN staining, MCAO induced severe neuronal cell death in the cortex on Day 3 (Fig. 3), and TubA, given immediately or at 24 hours after ischemia, significantly improved neuronal survival. Hence, the treatment time window of TubA was up to 24 hours after the initiation of the ischemia. For subsequent experiments, TubA was injected immediately after the onset of ischemia to achieve the maximal beneficial effects which may better enable discovery of potential underlying mechanisms.

### TubA restored α-tubulin acetylation levels and increased FGF-21 expression and signaling in ischemic brain

**Figure 4:**
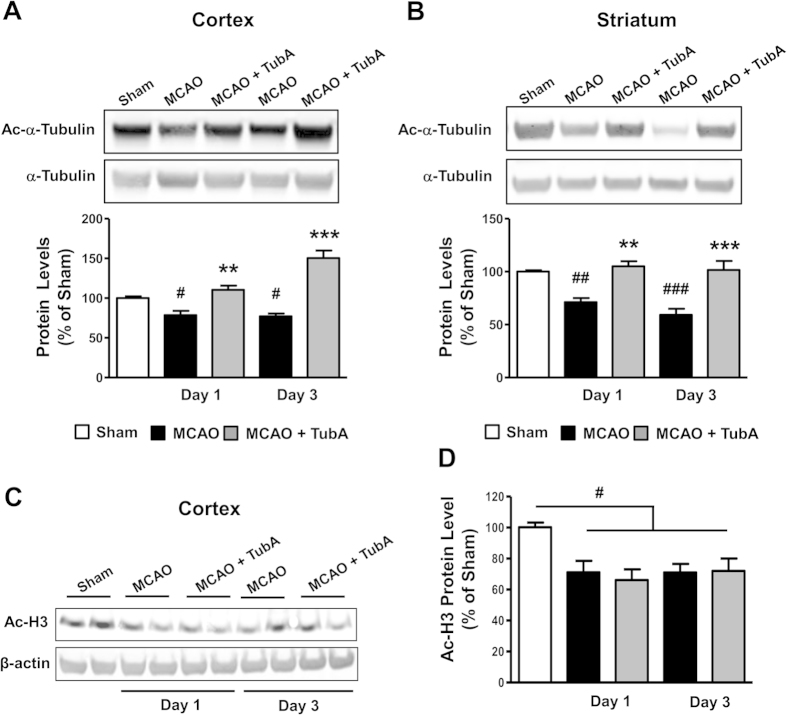
TubA (25 mg/kg) restored α-tubulin acetylation levels in the ischemic cortex and striatum on Days 1 and 3 after ischemia. Middle cerebral artery occlusion (MCAO) reduced the levels of acetylated α-tubulin (Ac-α-Tubulin) in the ischemic cortex (**A**) and striatum (**B**) on Days 1 and 3 after ischemia. TubA significantly increased α-tubulin acetylation levels. (**C**) TubA had no effect on restoring acetylated histone H3 (Ac-H3) protein levels in the cortex on Days 1 and 3 after ischemia. Quantified data is shown in (**D**). ^#^*P* < 0.05, ^##^*P* < 0.01, ^###^*P* < 0.001 compared with sham control; **P < 0.01, ***P < 0.001 compared with MCAO group; n = 6 per group (for α-tubulin acetylation); n = 4 per group (for histone H3 acetylation).

As a major substrate of HDAC6, increased α-tubulin acetylation indicates HDAC6 inhibition. The acetylation levels of α-tubulin were significantly reduced in both ischemic cortex and striatum of MCAO rats on Days 1 and 3 after ischemia compared with sham control (Fig. 4A,B). As expected, TubA restored α-tubulin acetylation levels to, or even beyond (cortex, Day 3) sham control levels in both ischemic brain regions but did not affect acetylated histone H3 levels (Fig. 4C,D), supporting that TubA does not non-selectively inhibit HDACs.

**Figure 5:**
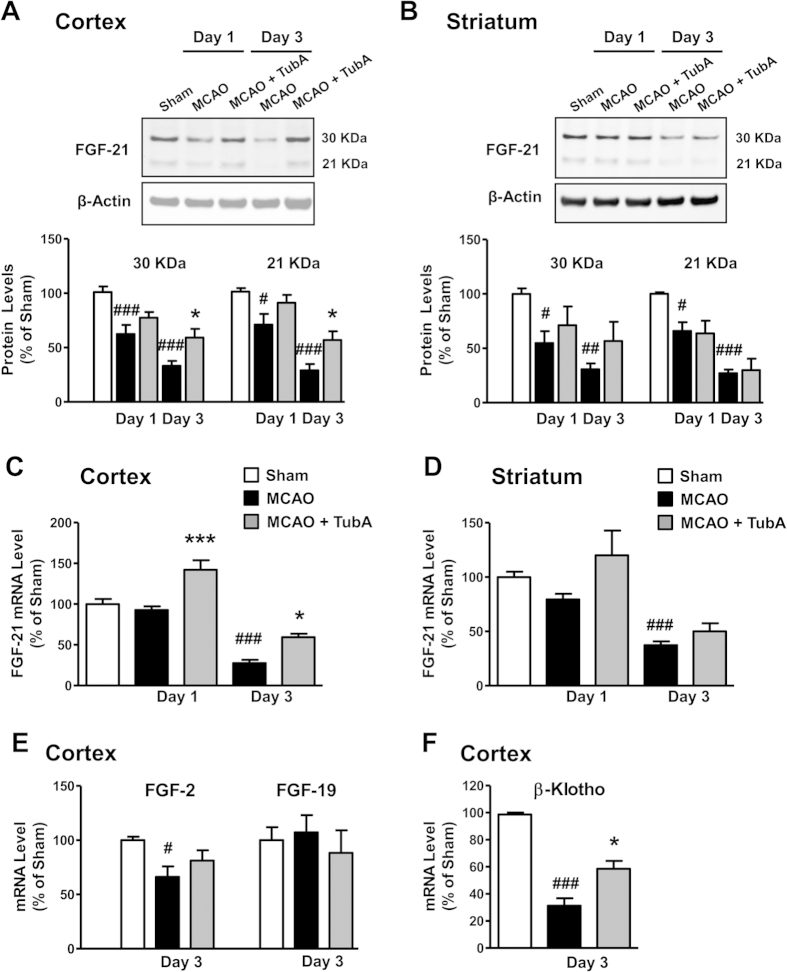
TubA (25 mg/kg) increased fibroblast growth factor-21 (FGF-21) levels in the ischemic cortex and striatum on Days 1 and 3 after ischemia. Middle cerebral artery occlusion (MCAO) reduced protein and mRNA levels of FGF-21 in the ischemic cortex (**A**) and striatum (**B**) on Days 1 and 3 after ischemia. FGF-21 mRNA levels were also markedly decreased in the ischemic cortex (**C**) and striatum (**D**) on Day 3. TubA significantly restored FGF-21 protein and mRNA levels on Day 3 in the cortex. FGF-2 and FGF-19 mRNA levels were not affected by TubA treatment in the ischemic cortex on Day 3 (**E**). β-Klotho mRNA levels were robustly decreased in the ischemic cortex on Day 3, and this decrease was markedly reversed by TubA treatment (**F**). ^#^*P* < 0.05, ^##^*P* < 0.01, ^###^*P* < 0.001 compared with sham control; *P < 0.05, ***P < 0.001 compared with MCAO group; n = 6 per group.

Because of the recently discovered neuroprotective properties of FGF-21, we investigated the involvement of this novel growth factor in the MCAO-induced pathophysiology and treatment effects of TubA. Two FGF-21 bands at 30 and 21 kDa were detected in both the cortex and striatum by Western blotting (Fig. 5A,B). Compared with the sham group, MCAO significantly decreased FGF-21 protein levels in the ischemic cortex and striatum on Day 1, and further declines were observed on Day 3. TubA treatment markedly increased FGF-21 protein levels in the ischemic cortex on Day 3, but did not significantly restore levels in the ischemic striatum. FGF-21 mRNA levels were also dramatically decreased in both cortex and striatum on Day 3 after ischemia (Fig. 5C,D). Consistent with changes in protein levels, TubA significantly increased FGF-21 mRNA levels in ischemic cortex rather than striatum. Interestingly, FGF-21 mRNA levels were not affected by MCAO in either the cortex or striatum on Day 1 compared with sham control, and these changes were inconsistent with those in protein levels. TubA’s effects on other FGFs were also evaluated. FGF-2 is a well-known neuroprotector, and FGF-19 and FGF-21 belong to the same subfamily of endocrine FGFs. MCAO significantly reduced FGF-2 mRNA levels in ischemic cortex on Day 3, but did not affect FGF-19 (Fig. 5E). TubA did not change either FGF-2 or FGF-19 mRNA levels in ischemic cortex, suggesting the selective effect of TubA on FGF-21.

**Figure 6:**
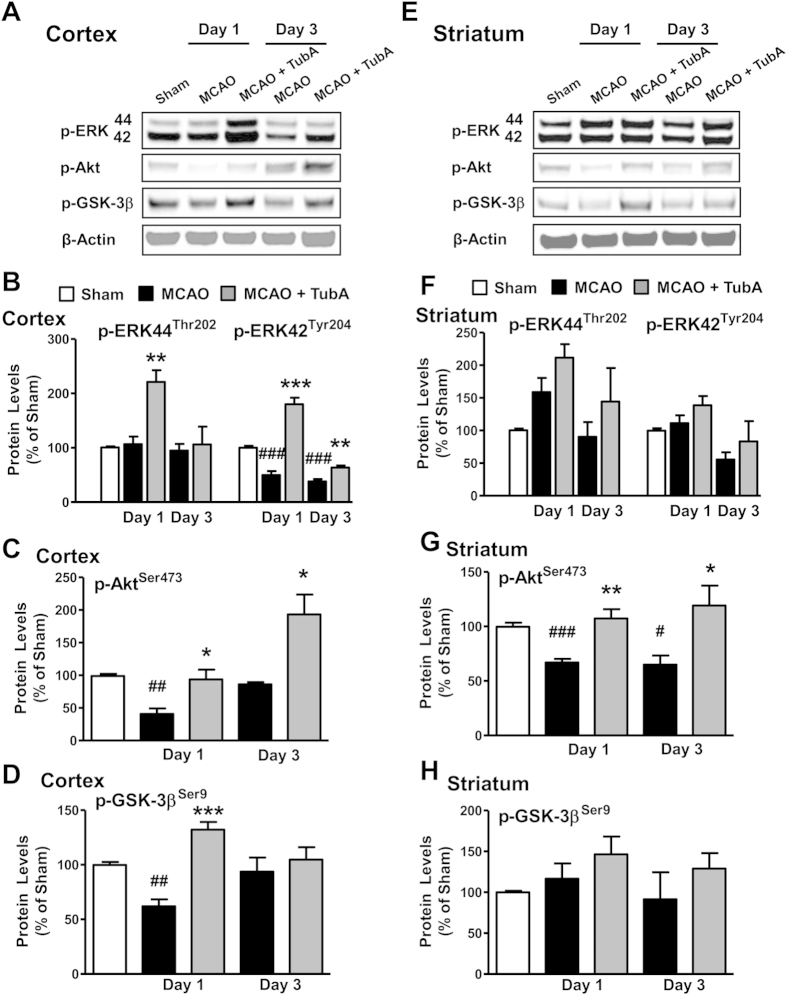
TubA (25 mg/kg) activated ERK and Akt/GSK-3β signaling pathways in the ischemic hemisphere on Days 1 and 3 after ischemia. Middle cerebral artery occlusion (MCAO) induced reductions in levels of phospho-ERK42^Tyr204^, phospho-Akt^Ser473^, and phospho-GSK-3β^Ser9^ in the ischemic cortex, especially on Day 1 after ischemia (**A–D**). In the ischemic striatum, phospho-Akt^Ser473^ was significantly reduced on Days 1 and 3, but levels of phospho-ERK44/42^Thr202/Tyr204^ and phospho-GSK-3β^Ser9^ were not affected (**E–H**). TubA significantly restored MCAO-reduced levels of phosphorylated ERK42^Tyr204^, Akt^Ser473^ and GSK-3β^Ser9^ in the ischemic cortex and striatum on Days 1 and 3. ^#^*P* < 0.05, ^##^*P* < 0.01, ^###^*P* < 0.001 compared with sham control; *P < 0.05, **P < 0.01, ***P < 0.001 compared with MCAO group; n = 6 per group.

We further evaluated TubA’s effects on FGF-21 signaling. The mRNA levels of β-Klotho, a FGF receptor co-activator, were robustly reduced in the ischemic cortex on Day 3, and this reduction was significantly reversed by TubA treatment (Fig. 5F). Furthermore, TubA robustly increased phospho-ERK44/42^Thr202/Tyr204^ levels in the ischemic cortex on Day 1, and the increase in phospho-ERK42^Tyr204^ levels by TubA were still significant on Day 3 (Fig. 6A,B). TubA also tended to increase phospho-ERK44/42^Thr202/Tyr204^ levels in the ischemic striatum on Days 1 and 3 (Fig. 6E,F). MCAO significantly reduced phosphorylation levels of Akt^Ser473^ in the ischemic cortex on Day 1 (Fig. 6A,C) and striatum on Days 1 and 3 (Fig. 6E,G), and phosphorylation levels of GSK-3β^Ser9^ in ischemic cortex on Day 1 (Fig. 6A,D). These reductions were markedly reversed by TubA treatment, indicating activation of Akt and inhibition of GSK-3β. Effects of TubA on FGF-21 signaling, including up-regulation of β-Klotho and activation of ERK and Akt/GSK-3β after brain ischemia were consistent with its effects on FGF-21 mRNA and protein expression.

### TubA and exogenous FGF-21 ameliorated glutamate-induced excitotoxicity and mitochondrial transport deficits in neurites of rat cortical neurons

**Figure 7:**
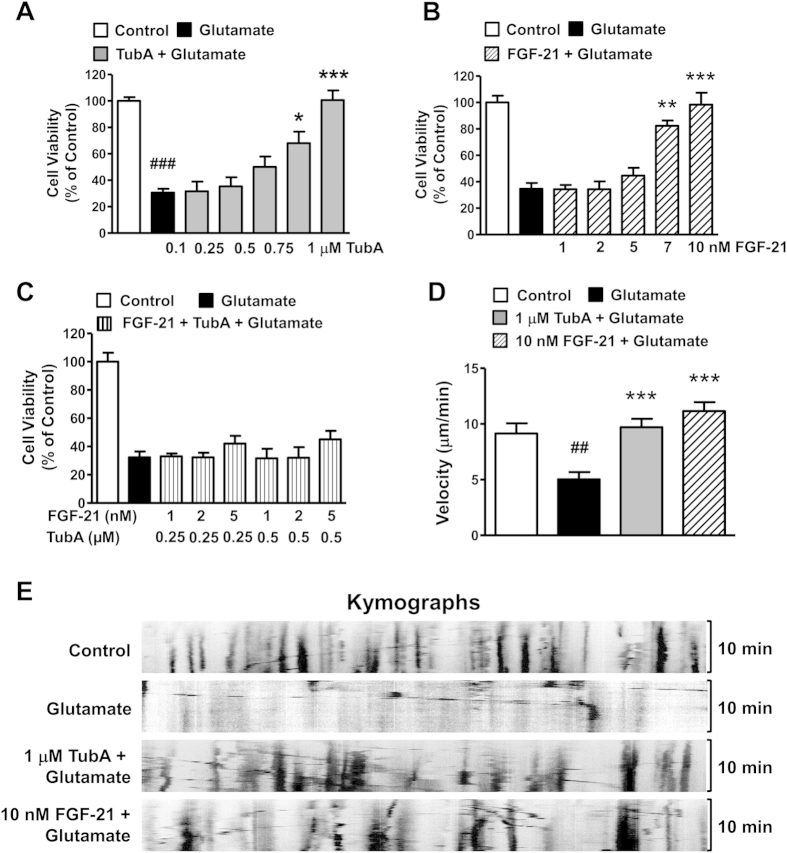
Pretreatment with TubA or recombinant human fibroblast growth factor-21 (FGF-21) protein protected cortical neurons from glutamate-induced excitotoxicity and mitochondrial transport deficits. TubA (**A**) or recombinant FGF-21 (**B**) protected cortical neurons against glutamate challenge in a dose-dependent manner. Combined treatment with sub-effective doses of TubA and FGF-21 did not have a synergistic effect (**C**). Average velocity of moving mitochondria (**D**) was significantly reduced after glutamate treatment, and was robustly restored by 1 μM TubA or 10 nM FGF-21 pre-treatment. (**E**) Representative kymographs of mitochondrial movement. ^##^*P* < 0.01, ^###^*P* < 0.001, compared with untreated control cells; **P<0.01, ***P < 0.001 compared with glutamate treated cells. For cell viability study, we employed n = 3; for mitochondria velocity analysis, 20 mitochondria were traced in each group and n = 3.

Given that glutamate overflow and excitotoxicity have been strongly implicated in stroke pathology, we used primary rat cortical neuronal cultures to assess the effects of TubA and FGF-21 on exogenous glutamate-induced excitotoxicity to explore any possible underlying protective mechanisms. In cortical neurons, 10 μM glutamate treatment markedly induced cell death, and pre-treatment with TubA or exogenous FGF-21 protected neurons from glutamate challenge in a dose-dependent manner (Fig. 7A,B). TubA at 1 µM or FGF-21 at 10 nM almost completely protected neurons from glutamate-induced cell death. We also evaluated the effects of combined pre-treatment with subeffective doses of TubA (0.25 and 0.5 μM) and FGF-21 (1, 2 and 5 nM) on glutamate-treated rat cortical neurons. However, no synergistic effect was observed on glutamate-induced cell death (Fig. 7C). To evaluate the effects of both TubA and FGF-21 on glutamate-induced mitochondrial transport deficits, we used live cell imaging to track MitoTracker Red-labeled mitochondria. TubA (1 μM) or FGF-21 (10 nM) pretreatment significantly ameliorated glutamate-impaired mitochondrial transportation in neurons (Fig. 7D). Kymographs showed that glutamate greatly reduced mitochondrial trafficking back and forth along the neurites, and TubA or FGF21 significantly increased the reduced mitochondrial movement due to the presence of glutamate (Fig. 7E).

## Discussion

This study demonstrated that the selective HDAC6 inhibitor TubA markedly reduced brain infarct volume, mitigated neuronal death, and improved functional deficits induced by focal cerebral ischemia in rats. These beneficial effects lasted for at least three days after ischemia. Strikingly, the time window of TubA treatment was at least 24 hours after the ischemic onset, which is substantially longer than that of other HDAC inhibitors including the pan inhibitor valproate in ischemic stroke models^18^. The robust neuroprotective effects and behavioral benefits in conjunction with the long time window suggest that TubA has promising utility for the treatment of ischemic stroke, underscoring the mounting evidence that selective inhibition of HDAC6 is neuroprotective and promotes regeneration after injury in the CNS^6^.

One of the important substrates of HDAC6 is α-tubulin, which forms dimers with β-tubulin in microtubules. In this study, we observed significant reductions in α-tubulin acetylation levels in both the cortex and striatum on Days 1 and 3 after cerebral ischemia. As expected, TubA treatment markedly restored levels of acetylated α-tubulin in the ischemic brain regions with no significant effect on overall histone acetylation levels, suggesting the selectivity of TubA on the HDAC6 isoform in our experimental conditions. Acetylation of α-tubulin can increase microtubule stability, enhance the interaction between α-tubulin and motor proteins, and facilitate microtubule-based transport^19^,^20^. Active microtubule-based transport of organelles, proteins, and RNAs is necessary for multiple neuronal processes, such as neurotransmission, axonal sprouting/regeneration, and synapse formation/retraction^21^. Axons and dendrites are vulnerable to cerebral ischemia, and the microtubule-based transport machinery within them is impaired after stroke^22^,^23^. Hence, strategies that restore impaired microtubule-based transport in axons and dendrites, for instance via HDAC6 inhibition, hold great promise for the treatment of stroke because of their putative ability to maintain the proper function of existing axons and to facilitate the consequent axon sprouting and synaptogenesis.

Mitochondria are prominent axonal microtubule-based transported organelles, and mitochondrial transport is usually considered as a general index for organelle axonal transport^24^. Mitochondria incur severe damage after cerebral ischemia that ultimately leads to cell death^25^. However, the impact of ischemia on mitochondrial transport is still unexplored. This study used glutamate-treated rat primary cortical neurons as an *in vitro* model to mimic cerebral ischemia-induced excitotoxicity. We found that more than half of cortical neurons degenerated after glutamate treatment, but that TubA almost completely prevented this glutamate-induced neuronal death. In addition, mitochondrial transport in the neurites of cortical neurons was markedly impacted by glutamate-induced excitotoxicity, but TubA treatment ameliorated this mitochondrial transport deficit. Taken together, our results suggest that TubA may improve cerebral ischemia-impaired mitochondrial transport, and the underlying mechanisms may involve enhanced microtubule stability via the up-regulation of α-tubulin acetylation.

FGF-21 is a circulating metabolic regulator of glucose and lipid metabolism^26^. It is mainly expressed in tissues with high metabolic activity and is generally induced by metabolic stress. It protects against diet-induced obesity and insulin resistance, and is significantly correlated with human liver disease^27^,^28^. Hence, FGF-21 has emerged as a promising treatment for obesity, type 2 diabetes, and liver diseases. Of note, very recent research from our laboratory found that brain neurons can express FGF-21, and that co-treatment with the mood stabilizers lithium and valproic acid can markedly increase its neuronal expression^17^. In addition, FGF-21 plays a robust neuroprotective role against glutamate challenge in rat cerebellar granular cells^17^. Because the role of FGF-21 in brain disorders is unknown, we examined change in FGF-21 expression in the brain after experimental cerebral ischemia and the effects of TubA on this regulation. Our results showed that FGF-21 protein levels were markedly reduced in the ischemic cortex and striatum, and that these reductions were restored by TubA treatment, especially in the cortex on Day 3 after ischemia. β-Klotho is a co-factor of the FGF receptor and is required for FGF-21 signaling^29^. Consistent with its effects on FGF-21 protein expression, TubA significantly up-regulated β-Klotho mRNA levels in the ischemic cortex on Day 3. ERK/MAPK signaling cascades can be activated by FGF-21 and participate in FGF-21’s protection against cardiac apoptosis induced by diabetes^30^,^31^. In the present study, TubA increased phosphorylation of ERK44/42 at Thr202/Tyr204, notably in the ischemic cortex, suggesting up-regulation of FGF-21 signaling after cerebral ischemia. Furthermore, in primary cortical cultures challenged with glutamate, exogenous FGF-21 had robust neuroprotective effects in preventing neuronal death and restoring mitochondrial neurite transport. These effects of FGF-21 echo those of TubA in primary neurons. Taken together, our findings suggest that FGF-21 may be neuroprotective against cerebral ischemia, and that TubA’s beneficial effects may be at least partially attributable to FGF-21 up-regulation.

There are three potential mechanisms by which TubA may up-regulate FGF-21 after cerebral ischemia. First, TubA may up-regulate FGF-21 through the Akt/GSK-3β signaling pathway, which has been shown to mediate FGF-21 induction by co-treatment with the mood stabilizers lithium and valproate in brain neurons^17^. A recent study found that TubA can activate Akt by enhancing Akt phosphorylation at Ser473 and inhibit GSK-3β by increasing phosphorylation of GSK-3β at Ser9 in the brains of Alzheimer’s disease mice^14^. In this study, we found that TubA also improved phosphorylation of Akt^Ser473^ and GSK-3β^Ser9^ in the ischemic rat brain. Therefore, by inhibiting HDAC6, TubA may activate Akt, inhibit GSK-3β, and consequently up-regulate FGF-21 in the ischemic brain.

Second, TubA may increase the intracellular transport of FGF-21 and its receptor. Microtubule network plays an essential role in intracellular protein trafficking. By increasing α-tubulin acetylation, HDAC6 inhibition facilitates BDNF transport and its subsequent release in experimental models of Huntington’s disease and Rett syndrome^8^,^16^. The secretion of FGF-21 likely also depends on transport within neurites, which is impaired by stroke. This may also explain why the decrease in FGF-21 protein levels preceded that of its mRNA levels after MCAO. Intracellular protein transport is driven by motor proteins kinesin and dynein. Loss of α-tubulin acetylation has been shown to influence the binding and motility of kinesin^20^, and the kinesin complex is responsible for the trafficking of the FGF receptor^32^. Accordingly, TubA may increase FGF-21 signaling through increasing α-tubulin acetylation, enhancing binding and motility of kinesin, and facilitating FGF receptor trafficking. Hence, it is possible that the trafficking and release of FGF-21 and its receptor is severely interrupted under ischemic conditions, and that treatment with TubA could reverse these deficits by inhibiting HDAC6, increasing α-tubulin acetylation, and subsequently facilitating microtubule-based transport.

Lastly, we found that TubA up-regulated FGF-21 mRNA levels as well as protein levels. Because HDAC6 is predominantly expressed in the cytoplasm and does not affect histone acetylation, TubA may affect FGF-21 mRNA transcription via indirect mechanisms. Given that Akt activation and GSK-3β inhibition are involved in FGF-21 transcription^17^, and that TubA can activate Akt and inhibit GSK-3β as mentioned previously, TubA may increase FGF-21 mRNA transcription via the Akt/GSK-3β pathway. HDAC6 has also been shown to associate with HDAC11, suggesting that HDAC6 may regulate gene expression through HDAC11^33^. Further investigation is required to identify the exact underlying mechanisms of TubA’s effects on FGF-21 transcription.

To our knowledge, this study is the first to demonstrate that TubA, most likely by inhibiting HDAC6, ameliorated mitochondrial transport deficits and preserved FGF-21 levels and signaling in the rat brain after cerebral ischemia, concurrent with robust decreases in infarction volume, neurological deficits and neuronal cell death. The therapeutic benefit of selective HDAC6 inhibition was not limited to improving microtubule-based transport by enhancing α-tubulin acetylation and microtubule stability. The underlying mechanisms may also include up-regulation in expression and signaling of FGF-21, a novel neuroprotective factor. Our findings suggest that HDAC6 is a promising target for the treatment of ischemic stroke, and clinical trials of TubA for this brain disorder are warranted.

## Methods

All animal experiments are reported in compliance with ARRIVE (Animal Research: Reporting *in Vivo* Experiments) guidelines where applicable. All experimental protocols were approved by the Animal Care and Use Committee of the National Institute of Mental Health at the National Institutes of Health.

### MCAO and drug administration

All animal experiments were performed according to protocols approved by the National Institute of Mental Health Animal Care and Use Committee. Male Sprague-Dawley rats (200–220 g, Charles River Laboratories, Wilmington, MA) underwent right middle cerebral artery occlusion (MCAO) under inhalational anesthesia (2.5% isoflurane in O_2_) as previously described^2^,^34^,^35^. TubA (provided by Dr. Alan Kozikowski, University of Illinois at Chicago) was first dissolved in dimethylsulfoxide (DMSO) and diluted with 2% Tween20 in phosphate buffered saline (PBS). The final concentration of DMSO was 5%. MCAO rats were randomized to TubA or vehicle treatment. TubA (25 or 40 mg/kg, i.p.) was injected immediately or 24 hours after ischemic onset, and then once daily for up to three days.

### Behavioral tests

The accelerating rotarod test, neurological deficit score, and elevated body tilting test were performed for three consecutive days after ischemia to assess the effects of TubA on the functional recovery of MCAO rats by an investigator blind to the treatment condition. Detailed procedures were previously described^35^. Briefly, for the accelerating rotarod test, rats were placed on an accelerating rotarod apparatus (San Diego Instruments, San Diego, CA), in which the speed was accelerated from 0 to 40 rpm over four minutes. Three consecutive days before MCAO, rats received once-daily training sessions of three trials separated by 30-minute intervals. The longest amount of time each rat remained on the rod was recorded as baseline. Three consecutive days after MCAO, rats underwent three trials on the rotarod, and the best performance of each rat was recorded for that day.

For the neurological deficit score, rats were assessed for motor, sensory, and reflex performance using a modified 12-point neurological scoring system. Seven tests of motor performance (flexion of forelimb, flexion of hind limb, head movement 10° to the vertical axis, inability to walk straight, circling towards the paralytic side, falling to the paralytic side, and immobility), two tests of sensation (visual and tactile placement and a proprioceptive test), and three reflex tests (pinna, corneal, and startle reflex) were evaluated. A score of 0 (normal) or 1 (abnormal) was given for each test.

Body asymmetry was quantitatively assessed using the elevated body tilting test. Rats were examined for lateral movements/turning when their bodies were suspended by the tail 200 mm above the testing table. The number of initial head or upper body turns was counted in 20 consecutive trials.

### Brain infarction measurement

Rats were sacrificed immediately after behavioral tests on Day 3 after ischemia, and the brains were quickly removed and placed on ice. TTC (2,3,5-triphenyl tetrazolium chloride, Sigma, St. Louis, MO) staining was performed to determine brain infarct volume, as previously described^36^. Briefly, six 2-mm coronal sections were stained with 2% TTC at 37 °C for 15 minutes and then fixed in 10% formalin overnight. The infarct area in white was measured using ImageJ software (free download at http://rsbweb.nih.gov/ij/). The infarct volume was calculated as the sum of infarct area times the average slice thickness (2 mm).

### Immunofluorescence staining

Brains were fixed with 10% formaldehyde by transcardial perfusion, dehydrated in 30% sucrose, and cryo-cut coronally at 30 μm. Free-floating sections were incubated with 0.5% Triton X-100 in PBS for 5 minutes, blocking solution (1% BSA with 0.05% Triton X-100) for 2 hours, and then overnight at 4 °C with mouse anti-NeuN (1:1,000, Millipore, Billerica, MA) in blocking solution. After washing, sections were incubated with Alexa Fluor® 488-conjugated secondary antibody (1:2000, Thermo Fisher Scientific, Frederick, MD). Immunolabeling signals were captured by a Zeiss AXIO Imager M2 microscope. ImageJ was used to quantify the results.

### Western blotting

The protein levels of acetylated α-tubulin, total α-tubulin, FGF-21, acetylated histone H3, phospho-Akt^Ser473^, phospho-GSK-3β^Ser9^, phospho-ERK44/42^Thr202/Tyr204^, and β-actin were detected by Western blotting in the ipsilateral cortex and striatum from Days 1 and 3 after ischemia. Briefly, brain tissue was dissected and sonicated in T-PER tissue protein extraction reagent (Thermo Fisher Scientific) containing protease and phosphatase inhibitor cocktails (Roche Diagnostics, Indianapolis, IN). The lysates were centrifuged at 12,000 rpm for 10 minutes at 4 °C and the supernatants were used for immunoblotting. Protein concentrations were determined using the BCA method (Thermo Fisher Scientific). Samples with equal total protein (1 μg/μl) were separated on a 4–12% Nupage Bis-Tris gel (Thermo Fisher Scientific) and transferred onto a nitrocellulose membrane (Thermo Fisher Scientific). The blots were blocked and incubated overnight at 4 °C with rabbit anti-acetyl-α-tubulin (1:5000, Thermo Fisher Scientific), rabbit anti-α-tubulin (1:3000, Cell Signaling Technology, Danvers, MA), rabbit anti-HDAC6 (1:2000, Cell Signaling Technology), rabbit anti-FGF-21 (1:1000, Aviscera Bioscience, Santa Clara, CA), rabbit anti-acetyl-histone-H3 (1:2000, Millipore, Billerica, MA), rabbit anti-phospho-Akt^Ser473^ (1:1000, Cell Signaling Technology), rabbit anti-phospho-GSK-3β^Ser9^ (1:1000, Cell Signaling Technology), rabbit anti-phospho-ERK44/42^Thr202/Tyr204^ (1:3000, Cell Signaling Technology), or mouse anti-β-actin (1:10,000, Sigma) in blocking buffer (LI-COR, Lincoln, NE) with 0.1% Tween 20. After washing, the membranes were incubated with appropriate IRdye 680 or 800CW conjugated secondary antibodies (1:10,000, LI-COR) for one hour at room temperature. Blotted proteins were detected and quantified using the Odyssey infrared imaging system (LI-COR).

### RNA extraction and real-time quantitative polymerase chain reaction

Total RNA was extracted from brain tissue using an RNeasy Mini kit (Qiagen, Valencia, CA) as per the manufacturer’s instructions. cDNA was reverse-transcribed from 2 μg of total RNA for each sample using a High-Capacity cDNA Reverse Transcription Kit (Thermo Fisher Scientific) according to the provided protocols. Real-time PCR reactions were conducted with FastStart Universal Probe Master (Roche Diagnostics) as well as with pre-made PrimeTime qPCR assays for FGF-21, FGF-2, FGF-19 or β-actin (Integrated DNA Technologies, Coralville, IA), or TaqMan gene expression assays for β-Klotho or β-actin (Thermo Fisher Scientific) using a 7500 Real Time PCR System (Thermo Fisher Scientific). mRNA levels of these genes were normalized against those of β-actin and presented as 2^−ΔΔCT^.

### Primary rat cortical neuronal culture

The brains of 18-day rat embryos were used to prepare cerebral cortical neuronal cells, as previously described with modifications^17^. Briefly, cortices were dissected from the embryonic brain. Cells were mechanically dissociated in Ca^2+^ and Mg^2+^-free HBSS buffer (Thermo Fisher Scientific) and then incubated in this HBSS buffer for 12 minutes; the buffer was replaced every three minutes. The disassociated cells were resuspended in 10% FBS DMEM medium at a density of 5 × 10^5^ cells per ml on six-well plates with or without cover slides precoated with 0.05% poly-D-lysine. After 24 hours, the culture medium was replaced with 2% serum-free B27 (Thermo Fisher Scientific)/Neurobasal Medium (Thermo Fisher Scientific) with 1 μM cytosine arabinoside to arrest the replication of non-neuronal cells.

### TubA and exogenous FGF-21 treatment, glutamate challenge, and cell viability measurement in primary cortical neurons

Starting at seven days *in vitro* (DIV-7), cortical neurons were pre-treated with 0.1, 0.25, 0.5, 0.75 and 1 μM TubA for two days, and 10 μM glutamate was added at DIV-9. Cell viability was assessed using a Cell Counting Kit-8 (Dojindo Molecular Technologies, Rockville, MD) 24 hours after glutamate treatment. Cell viability results were expressed as percentage of the vehicle-treated control.

Starting at DIV-2, cortical neurons were pre-treated with 1, 2, 5, 7 and 10 nM recombinant human FGF-21 protein (PeproTech, Rocky Hill, NJ) for seven days, and 10 μM glutamate was added at DIV-9. Cell viability was assayed 24 hours later using a Cell Counting Kit-8 (Dojindo Molecular Technologies).

To test whether there is a synergistic effect by combined pre-treatment with TubA and FGF-21, starting at DIV-2, cortical neurons were pre-treated with 1, 2, or 5 nM recombinant human FGF-21 protein for five days, and at DIV-7, 0.25 or 0.5 μM of TubA was added. At DIV-9, 10 μM glutamate was added for 24 hours. Cell viability was evaluated by using a Cell Counting Kit-8 (Dojindo Molecular Technologies).

### Live cell imaging and imaging analysis

Primary cortical neurons were cultured on 25 mm glass coverslips coated with 0.05% poly-D-lysine. The cell-attached coverslip was secured to a slide chamber filled with cell culture medium from the original culture well. Live cells were imaged using an Olympus IX81 reverse wide-field microscope using a 60 × 1.45 NA oil objective. During recording, cells were maintained in a stage incubating chamber at 37 °C. Time-lapse image recordings were acquired at an exposure time of 200 ms per frame, at 10-second intervals over a period of 10 minutes by MetaMorph Image Analysis software (Molecular Device, Sunnyvale, CA). The kymographs of mitochondria motility (time/distance re-slicing) were generated and analyzed by Fiji (a distribution of Image J for life sciences, NIH).

### Statistical analyses

Data are expressed as mean ± SEM. For behavioral data, two-way repeated-measures analysis of variance was performed to analyze the overall difference between treatment groups over time. Bonferroni-corrected post-hoc comparisons were then used to analyze the difference between groups at each time point. Comparisons between two groups and multiple groups were evaluated by Student’s *t*-test and one-way analysis of variance followed by Tukey’s post-hoc comparisons, respectively. Differences were considered statistically significant at *P* < 0.05.

## Additional Information

**How to cite this article**: Wang, Z. *et al.* Tubastatin A, an HDAC6 inhibitor, alleviates stroke-induced brain infarction and functional deficits: potential roles of a-tubulin acetylation and FGF-21 up-regulation. *Sci. Rep.* **6**, 19626; doi: 10.1038/srep19626 (2016).
